# Delay in Diagnosis of Classical Homocystinuria

**DOI:** 10.1002/jmd2.70087

**Published:** 2026-06-03

**Authors:** Subadra Wanninayake, Reena Sharma, Diane Green, John Bassett, Tarekegn Geberhiwot, Charlotte Dawson

**Affiliations:** ^1^ Department of Inherited Metabolic Disorders Queen Elizabeth Hospital Birmingham Birmingham UK; ^2^ Adult Inherited Metabolic Diseases, Salford, Care Organization, Northern Care Alliance Salford UK; ^3^ University of Manchester Manchester UK; ^4^ Tameside and Glossop Integrated Care NHS Foundation Trust, Ashton‐under‐Lyne Lancashire UK; ^5^ Institute of Metabolism and System Research University of Birmingham Birmingham UK

**Keywords:** CBS, classical homocystinuria, delay in diagnosis, diagnostic gap

## Abstract

Classical homocystinuria (HCU) is an autosomal recessive disorder of methionine metabolism with a wide spectrum of severity and clinical presentation. Timely diagnosis facilitates prompt initiation of treatment, which reduces complications. Our aim was to identify the nature of the first clinical manifestation and time to subsequent diagnosis in our cohort of adults with HCU. This retrospective cross‐sectional study was conducted in two tertiary referral centres for adult inherited metabolic disorders in the United Kingdom. Fifty‐nine patients with sufficient clinical data for detailed analysis were included. 13/59 patients were detected asymptomatically through newborn or family screening and 46/59 were diagnosed on initial presentation with a clinical manifestation of HCU. For 15/54 (27.8%), the median time between initial presentation and diagnosis was 7 years (IQR, 2–11.9), the commonest first manifestation in the delayed group was lens subluxation (6/15, 40%) followed by venous thromboembolism (5/15, 33.3%) and skeletal deformities (2/15, 13.3%). 15/46 (32.6%) had two or more complications by the time of diagnosis. Lens subluxation is the commonest first manifestation of HCU in the group with delayed diagnosis. Early recognition, expanded screening and enhanced clinician awareness are essential for timely diagnosis and improved outcomes.

## Key Points

- Pyridoxine‐responsive and partially pyridoxine‐responsive classical HCU is not detected on newborn screening and presents later in childhood or in adulthood.
- Most common presenting features are rapid onset myopia caused by lens subluxation followed by potentially life‐threatening venous thromboembolism.
- One‐third of patients with pyridoxine‐responsive and partially pyridoxine‐responsive forms were not diagnosed at initial presentation and in this cohort there was a mean diagnostic delay of 7 years between first clinical manifestation and final diagnosis of HCU.
- Two‐thirds of this cohort had two or more complications at the time of diagnosis.

**Abbreviations**
CVT: cerebral venous thrombosis
HCU: classical homocystinuria
Hcy: homocysteine
Met: methionine
tHcy: total homocysteine

## 1 Background

Classical homocystinuria (HCU, OMIM #236200) is a rare autosomal recessive disorder caused by deficiency of cystathionine beta‐synthase (CBS), an enzyme responsible for converting homocysteine (Hcy) to cystathionine in methionine (Met) metabolism [1]. CBS deficiency results in elevated plasma and tissue levels of Hcy and Met, affecting multiple tissues, especially the liver, heart, skeletal and nervous systems [1, 2, 3]. Clinical presentations vary widely, from severe multi‐systemic disease in childhood to asymptomatic adulthood, with common features including ophthalmological abnormalities (myopia, ectopia lentis), neurological impairment (developmental delay/intellectual disability), skeletal deformities (marfanoid habitus, scoliosis and pectus excavatum) and thromboembolic events [1].

Global prevalence is estimated between 1:200 000 and 1:335 000, with UK incidence around 1:60 000 to 1:100 000, varying by ethnicity [4, 5]. The actual prevalence of HCU may be underestimated because many cases remain undiagnosed, often presenting later with vascular or ocular complications [6, 7, 8, 9]. Reports in the late 1990s indicate a considerable delay between symptom onset and diagnosis, a gap still evident in countries lacking screening programmes, which may have a devastating impact on patients [7, 8, 10, 11, 12]. We observed the extent of diagnostic delay remains in the United Kingdom. Despite the UK's advanced diagnostics and inclusion of HCU in the newborn screening programme since 2014, NBS uses raised Met as a primary test to identify patients with HCU and its sensitivity is limited, particularly for pyridoxine ‐responsive CBS deficiency [1]. This study aims to understand the scale of the gap in the United Kingdom in the early 21st century by measuring the nature of the first clinical manifestation and time taken to subsequent diagnosis.

## 2 Methods

This retrospective cross‐sectional study was conducted in two regional, Inherited Metabolic Disease (IMD) clinics in the United Kingdom for adult patients; at the University Hospitals Birmingham NHS FT (UHB) and Northern Care Alliance NHS FT. This study was conducted subsequent to formal clinical audit registration as a part of service evaluation at UHB and with the local Research and Development (R and D) team as a health improvement project at Northern Care Alliance NHS FT. We undertook a search on the medical records on the trust electronic system of all patients with HCU, who were diagnosed based on clinical symptoms followed by clear elevations in Hcy and Met, since their management started at the IMD clinic. Patients with incomplete data related to their first clinical manifestation and leading cause for diagnosis of HCU on the hospital records were excluded.

For the purposes of this study, participants were categorised into three groups according to their response to the pyridoxine (Vitamin B6) challenge test, which involved administering 500 mg/day of pyridoxine and measuring changes in plasma tHcy:

Responsive: Marked improvement with tHcy falling below 50 μmol/L on pyridoxine alone.

Partially responsive: At least a 20% reduction in tHcy, but levels remained elevated and required additional therapy.

Non responsive: Less than a 20% reduction in tHcy post‐pyridoxine challenge.

## 3 Statistical Analysis

Statistical analyses were conducted using Microsoft Excel. Descriptive statistical analysis was done. Continuous variables were expressed as mean and standard deviation (SD) for parametric data and median with interquartile range (IQR) for non‐parametric data. Categorical variables were summarised in terms of number, frequency and percentage.

## 4 Results

**FIGURE 1:**
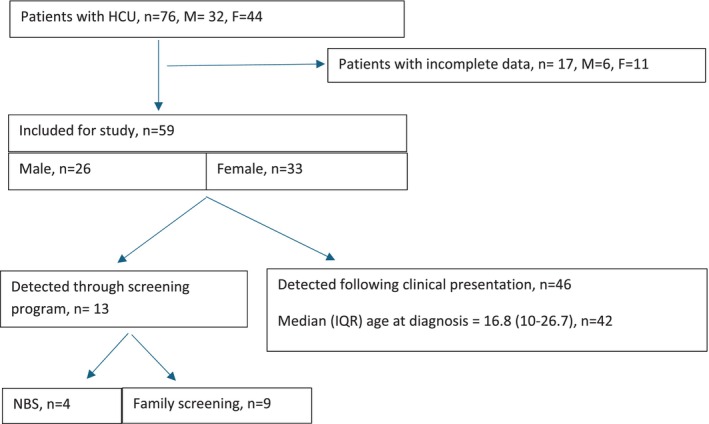
Summary of the patients included into the study.

A total of 76 patients with diagnosed HCU, followed at two institutions serving an approximately 22 million of population at the time of data collection, were included in the analysis. The cohort comprised 32 males and 44 females, with a median age at assessment of 36.1 years (IQR 29.1–49.1, range 19.4–83.3). Demographic characteristics, clinical features and complications HCU of the cohort are summarised in Table 1. Of these, 59 patient records contained sufficient clinical data for detailed analysis concerning clinical presentation and/or timing of diagnosis; in 17 cases, clinical symptoms or age at presentation were insufficiently documented (Figure 1).

**TABLE 1:** Demographic data, clinical characteristics and complications of HCU of the cohort (*n* = 76).

Parameter	Median (IQR)/number of patient (%)
Age	36.1 (29.1–49.1)
Male/female	32/44
Type of HCU	
Pyridoxine‐responsive	28
Pyridoxine non‐responsive	22
Partially pyridoxine‐responsive	13
Unknown	13
Clinical characteristic	
Eye involvement	44/52 (84.6%)
Thromboembolism	29/45
Marfanoid features	4/11
Osteoporosis	11/25
Skeletal deformities	34/34
Developmental delay	4/36
Learning difficulties	14/36
Behavioural problem	9/36
Depression	10/34
Laboratory findings at baseline	
tHCy	107 (46–179)/*n* = 53
Methionine	135 (62–235)/*n* = 8
RBC count	4.6 × 10^12^/L (4.3–4.9)/*n* = 20
Treatment (*n* = 63)	
On pyridoxine	*n* = 40
Betaine	*n* = 23

### 4.1 Clinical Presentations Leading to Diagnosis and Complications Prior to Diagnosis

**FIGURE 2:**
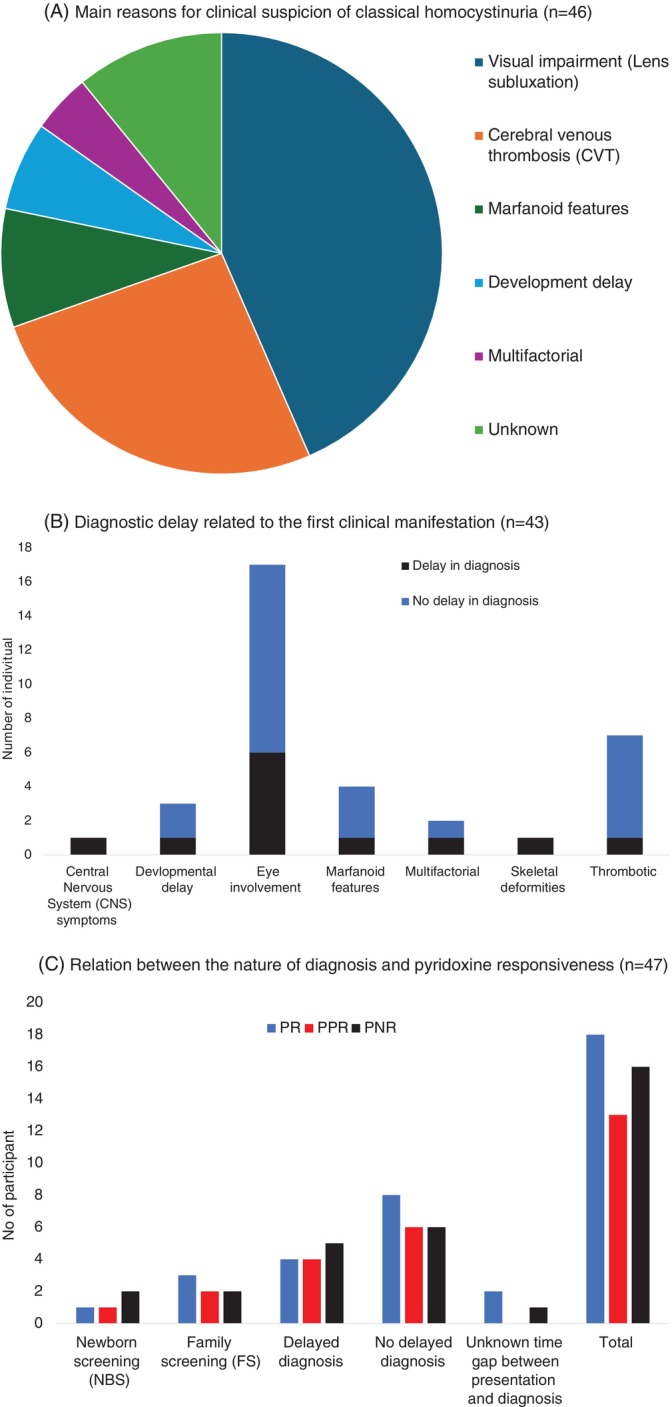
(A) Main reasons for clinical suspicion of classical homocystinuria (*n* = 46). (B) Diagnostic delay related to the first clinical manifestation (*n* = 43). (C) Relation between the nature of diagnosis and pyridoxine responsiveness (*n* = 47).

Figures 1 and 2 show the primary clinical manifestations prompting HCU diagnosis. Of the 59 patients with detailed records, 46 (78%) were diagnosed following clinical presentation. Ocular manifestations were noted in 26/46 at the time of diagnosis. For the patients who diagnosed following clinical presentation, the median age at diagnosis was 16.8 years (IQR; 10–26.7, *n* = 42). The median age of first clinical symptoms (*n* = 43) was 12 years (IQR, 6.5–24.2). Of those, 27/46 patients were diagnosed at their initial clinical presentation and records of 4 patients did not have data to identify the time gap between clinical presentation and the diagnosis. For the remaining 15/46 (32.6%) patients, the median delay between initial clinical manifestation and diagnosis was 7 years (IQR, 2–11.9, range 0.1–25.6). The most common initial manifestation among these delayed cases were lens subluxation (6/15), followed by venous thromboembolism (5/15) and skeletal deformities (2/15) (Figure 2B). The common complications due to high Hcy levels before and after the diagnosis of HCU, in participant who had delay in diagnosis, is summarised on Table 2. Notably, 9/15 had two or more complications by the time of diagnosis (Table 2). The mean age at first clinical manifestation in patients diagnosed clinically (*n* = 42) was 15 years (SD 10.9, range 0.1–38). Their total homocysteine (tHcy) at diagnosis is > 200 μmol/L for the majority of the patients (Table 2). Additionally, nine patients were identified via family screening, and four through newborn screening.

**TABLE 2:** Clinical characteristics of the participants with delayed diagnosis (*n* = 15).

No.	Gender	Age (years)	Age at first symptoms (years)	Age at diagnosis (years)	Delayed in diagnosis (years)	Leading cause for diagnosis	First symptoms	Other complications before diagnosis	tHcy at diagnosis	Plasma methionine	Type of HCU	Complications after diagnosis
1	M	25.2	12.0	17.2	5.3	Visual impairment (mainly lens subluxation)	Eye involvement	Poor eyesight (since age 12 years), B/L lens dislocation (15 years).	308		PPR	
2	F	32.5	23.3	23.9	0.7			B/L ectopia lentis	296	61	PR	Scoliosis, seizures, depression, CVT in 2 weeks of postpartum
3	M	31.2	25.4	29.5	4.1			B/L blurred vision due to progressive myopia, then B/L lens subluxation, sagittal sinus thrombosis and seizures (26 years), stroke—venous thrombosis (29 years)	> 350	61	PR	Seizures
4	F	31.0	4.0	11.0	7.0			Myopia and abnormal gaits (4 years). Progressively worsening of eyesight over the next few years.	323	> Upper reference limit	PNR	Dislocated lens, osteoporosis with multiple fractures, kyphoscoliosis, learning difficulties, left internal jugular vein thrombosis, deep venous thrombosis of leg, pulmonary embolism.
5	F	33.6	10.6	21.6	11.0			Visual problems started and continued, dislocated lenses (11 years), pectus carinatum 1.5 years, speech delayed was reported.	NK	553	PNR	Marfanoid features, scoliosis
6	M	19.4	3.0	15.9	12.9		CNS	Cerebral palsy (3 years), Increase lens size (detected at 5 years), dramatically increased over 2 years, Marfan syndrome	315		PNR	B/L dislocated lenses, scoliosis, and pectus carinatum
7	M	45.6	34.6	36.6	2.0	CVT	CVT	Second cerebral venous thrombosis, no eye involvement	331	58	PPR	
8	M	38.1	20.1	27.1	7.0	Sagittal sinus thrombosis (20 years), lenses dislocation, osteoporosis, R/cranial interval artery thrombosis (27.1 years)	341	234	PR	
9	F	42.1	30.1	31.1	1.0	CVT—cortical vein thrombosis, raised MCV, PE	> 350		PR	B/L dislocated lenses, equinovarus deformity, presence of multiple seizures
10	F	39.9	19.9	21.9	2.0	DVT (19.9 years) lead to further diagnosis and identified in age of 21.9 years	NK			B/L dislocated lenses, multiple early miscarriages, panic attacks, idiopathic intracranial hypertension (at 33 years)
11	F	20.6	11.0	11.1	0.1	Intracranial venous thrombosis (11 years) diagnosis withing 1 month, presence of seizures	NK		PNR	B/L dislocated lenses, tall stature, learning difficulty, challenging behaviour, autistic spectrum disorder
12	M	35.6	7.0	32.6	25.6	Skeletal deformities	At 7 years, legs give way and weakness in legs and muscle wasting. Diagnosed with Charcot–Marie–Tooth disease, surgeries for Achilles tendon and high‐arched feet, cognitive problems and learning difficulties following meningitis, facial dysmorphism, wide‐spaced eyes, saddle nose deformity, bilateral pes cavus and bilateral hammertoes, small feet, TIA	125	16	NK	
13	F	20.5	5.0	14.7	9.7	Fits	Eye involvement	Lense dislocation and seizure at age of 5 years, developmental delay, MRI—chronic bilateral cerebellar infarcts with cerebellar volume loss.	> 250		PPR	Mild to moderate learning disability
14	M	48.5	0.5	13.5	13.0	Multifactorial	Developmental delay	At 5.5 month, easy distractible, clumsy and needed some speech therapy. Poor vision (6.5 years), dislocated lens removal (8.5 years). Learning disabilities	55	352	PNR	Osteoporosis, spastic paraparesis
15	F	46.1	12.1	26.1	14.0	Marfan's syndrome	Marfanoid features	BL, since birth, Marfan's syndrome diagnosed aged of 12, CVT (20 years)	200		PPR	

Abbreviations: B/L, bilateral; CNS, central nervous system; CVT, cerebral venous thrombosis; F, female; HCU, homocystinuria; M, male; NK, unknown; PNR, pyridoxine non‐responsive; PPR, partial pyridoxine responsive; PR, pyridoxine responsive; tHcy, total homocysteine.

### 4.2 Pyridoxine Responsiveness and Diagnostic Correlations

Data on pyridoxine responsiveness were available for 47 of the 54 patients. Of those, 16 were pyridoxine non‐responsive (PN), 18 were pyridoxine responsive (PR) and 13 were partially pyridoxine responsive (PPR). Within the subgroup diagnosed following clinical presentation (*n* = 36), 12 (33%) were PNR, 14 (39%) PR and 10 PPR; data were unavailable for 6 patients. The distribution of pyridoxine responsiveness in relation to diagnostic modality is shown in Figure 2C. Statistical analysis revealed no significant difference in pyridoxine responsiveness between patients with delayed diagnosis and those diagnosed promptly (*χ* ^2^ = 0.3554, *p* = 0.55). Among patients with delayed diagnosis, 5/11 (45%) were PNR, 4/11 (36%) PPR and 4/11 (36%) PR.

## 5 Discussion

The diagnosis of HCU remains challenging, often resulting in irreversible and potentially fatal complications in untreated patients. In our cohort, 77.7% of diagnoses were made based on clinical manifestations, which is expected as many patients were born before the implementation of newborn screening (NBS) for HCU in the United Kingdom. Notably, among cases identified through cascade family screening, 12.5% had complications related to hyperhomocysteinaemia at diagnosis. This contrasts with previous reports where patients identified by screening were asymptomatic at detection [10], underscoring the critical importance of early recognition and diagnosis of this treatable disorder.

The median age of first clinical symptoms in our cohort was 12 years (range 0.1–38), which aligns closely with a previously reported mean age of 13 years from studies in the 1990s [10]. Although most patients presenting with clinical features were diagnosed at initial presentation, more than one‐third experienced significant diagnostic delays despite evident symptoms. The mean age at subsequent diagnosis in our study was 17.7 years, earlier than the 24‐year reported in existing literature [10]. The mean diagnostic delay of 7.7 years observed here falls between delays reported recently (5 years) [12] and those documented in earlier studies (11 years) [10].

With regards to pyridoxine responsiveness, Patients diagnosed without delay were predominantly pyridoxine‐responsive, while those with delayed diagnoses were more often pyridoxine non‐responsive or partially responsive. This finding is somewhat unexpected, as pyridoxine non‐responsive individuals typically manifest severe symptoms or developmental delays early in childhood if undiagnosed by newborn screening, whereas pyridoxine‐responsive patients often remain asymptomatic or present with milder symptoms [1, 6, 13].

We acknowledge that, as this study was conducted in only two institutions, caution is warranted when interpreting the analysis of pyridoxine responsiveness and diagnostic timing. However, these two large metabolic centres serve nearly one‐third of the UK population. The prevalence of HCU, based on the number of known patients, is approximately 0.35/100 000—within the range of reported estimates [14, 15] albeit at the lower end. This figure is notably lower than the estimated global prevalence derived from symptomatic diagnoses (0.82 per 100 000; 95% CI: 0.39–1.73) and newborn screening data (1.01 per 100 000; 95% CI: 0.34–3.55) [14, 15]. This discrepancy may reflect an overestimation of global prevalence in a systematic review and meta‐analysis due to variations in ethnicity and geographic distribution, with higher rates in certain populations skewing the overall estimate. Nevertheless, given that these two centres serve a highly diverse population, it remains plausible that undiagnosed cases exist within this cohort.

Ocular involvement, particularly lens subluxation, was the most common clinical manifestation (84.6%) and leading cause for diagnosis (47.6%) in our cohort, consistent with previous studies [10, 12]. Ophthalmological complications remain the predominant systemic presentation, with 62% of clinically diagnosed patients exhibiting ocular signs at diagnosis. Ectopia lentis is a key early sign of untreated HCU, affecting approximately 50% of patients by age 10 years and over 90% by age 24 years, typically presenting by age 8 years [12, 13, 16]. Rapidly progressing lenticular myopia before age 5 years is rare and should prompt consideration of metabolic disorders. Myopia can also present in HCU without ectopia lentis, generally after age 1 year, emphasizing the need for biochemical investigation before adolescence. Despite the increasing prevalence of myopia in the general paediatric population [17], clinicians should remain vigilant for underlying metabolic aetiologies. In our cohort, ocular involvement was more pronounced among pyridoxine non‐responsive patients, although the small sample size limits statistical significance; the literature suggests earlier ocular manifestations in non‐responsive individuals [1].

Thromboembolism was the second most common cause for diagnosis (28%), contradicting lower reported prevalence at diagnosis [13]. Of the eight patients who experienced cerebral venous thrombosis (CVT) prior to diagnosis, HCU was suspected in only five, with just one diagnosed without delay. Thromboembolic events, often cerebrovascular in nature, are a major cause of morbidity and early mortality, and may present in adults who were asymptomatic during childhood [1, 6, 18]. While CVT is more frequently seen in young adults, cases have been documented in childhood and infancy [13, 19, 20, 21, 22]. Most CVT patients in our cohort were partially pyridoxine‐responsive, consistent with literature describing CVT occurrences in pyridoxine‐responsive patients during adolescence or adulthood [9, 23]. Although none of our patients were diagnosed due to seizures, all those with CVT had experienced unprovoked seizures, which have been reported as a common early manifestation in other cohorts [12]. One patient had both miscarriage and postpartum deep vein thrombosis (DVT), highlighting the increased thromboembolic risk associated with pregnancy, particularly postpartum [24].

Neurological and skeletal manifestations were equally responsible for prompting diagnosis in our cohort. Developmental delay, intellectual disability and psychiatric conditions are commonly observed in pyridoxine non‐responsive individuals [1, 25]. Central nervous system (CNS) involvement was frequent in untreated patients, with learning difficulties, depression, psychiatric issues and developmental delay being common. Developmental delay was the second most common first clinical manifestation in Brazilian patients [12]. Skeletal abnormalities, though less common at diagnosis in our cohort, are often detectable later in childhood and include conditions such as scoliosis, high‐arched palate and pes cavus [1]. HCU patients are prone to osteoporosis, with 50% showing signs by their teens [26]. In our cohort, 11 of 25 patients had DEXA‐proven osteoporosis [1, 26].

In patients with delayed diagnosis, identification typically occurred following progressive worsening of existing symptoms, recurrent complications, or patients seeking advice from other healthcare professionals. Diagnostic delay can be attributed to the wide clinical heterogeneity of HCU, the absence of classical textbook presentations and insufficient awareness among primary care providers regarding the disease's manifestations and complications.

Early diagnosis is paramount to prevent serious complications such as life‐threatening thromboembolism, cognitive impairment and ectopia lentis [19, 27]. Implementation of NBS in the United Kingdom is expected to reduce the overall diagnostic delay but will predominantly benefit patients with pyridoxine‐non‐responsive HCU. This is because NBS follows a tiered approach using Met as the initial screening marker with plasma tHcy confirmation for elevated results. In pyridoxine‐responsive and partially‐responsive forms, Met is not sufficiently elevated to be detectable on NBS. Hence, clinicians must maintain vigilance about the possibility of HCU in children and adults presenting with clinical features of the condition and request plasma tHcy as a sensitive and cost‐effective diagnostic test. Updating clinical guidelines to promote measurement of plasma tHcy in suspected cases of pyridoxine‐responsive and partially‐responsive forms would help to facilitate timely diagnosis in this cohort.

## 6 Conclusions

Delayed diagnosis of HCU remains common, contributing to preventable complications. Lens subluxation was the commonest clinical presentation and first manifestation of HCU in the group with delayed diagnosis. Tiered NBS will continue to enable pre‐symptomatic diagnosis of non‐pyridoxine‐responsive HCU. In other forms, early clinical recognition and measurement of plasma tHcy is essential for timely diagnosis and improved outcomes. Targeted education to increase awareness and updated guidelines to recommend requesting tHcy where there is a high index of clinical suspicion would facilitate timely diagnosis in pyridoxine‐responsive and partially‐responsive forms of the condition that will not be diagnosed in newborn screening programmes utilising Met as the primary biomarker.

## Author Contributions

S.W., C.D., T.H. and R.S. conceived the study. S.W., R.S., D.G. and J.B. involved in data collection and drafted the manuscript. All authors made contributions to writing and reviewing of the final manuscript.

## Funding

The authors have nothing to report.

## Ethics Statement

The authors have nothing to report.

## Consent

The authors have nothing to report.

## Conflicts of Interest

The authors declare no conflicts of interest.

## Data Availability

The data that support the findings of this study are available from the corresponding author upon reasonable request.
